# *Helicobacter pylori* chemoreceptor TlpC mediates chemotaxis to lactate

**DOI:** 10.1038/s41598-017-14372-2

**Published:** 2017-10-26

**Authors:** Mayra A. Machuca, Kevin S. Johnson, Yu C. Liu, David L. Steer, Karen M. Ottemann, Anna Roujeinikova

**Affiliations:** 1 Infection and Immunity Program, Monash Biomedicine Discovery Institute, Monash University, Clayton, Victoria, 3800 Australia; 2 Department of Microbiology, Monash University, Clayton, Victoria, 3800 Australia; 3 Department of Microbiology and Environmental Toxicology, University of California Santa Cruz, Santa Cruz, CA 95064 USA; 4 Monash Biomedical Proteomics Facility, Monash University, Clayton, Victoria, 3800 Australia; 5 Department of Biochemistry and Molecular Biology, Monash University, Clayton, Victoria, 3800 Australia

## Abstract

It is recently appreciated that many bacterial chemoreceptors have ligand-binding domains (LBD) of the dCACHE family, a structure with two PAS-like subdomains, one membrane-proximal and the other membrane-distal. Previous studies had implicated only the membrane-distal subdomain in ligand recognition. Here, we report the 2.2 Å resolution crystal structure of dCACHE LBD of the *Helicobacter pylori* chemoreceptor TlpC. *H*. *pylori tlpC* mutants are outcompeted by wild type during stomach colonisation, but no ligands had been mapped to this receptor. The TlpC dCACHE LBD has two PAS-like subdomains, as predicted. The membrane-distal one possesses a long groove instead of a small, well-defined pocket. The membrane-proximal subdomain, in contrast, had a well-delineated pocket with a small molecule that we identified as lactate. We confirmed that amino acid residues making contact with the ligand in the crystal structure—N213, I218 and Y285 and Y249—were required for lactate binding. We determined that lactate is an *H*. *pylori* chemoattractant that is sensed via TlpC with a *K*_D_ = 155 µM. Lactate is utilised by *H*. *pylori*, and our work suggests that this pathogen seeks out lactate using chemotaxis. Furthermore, our work suggests that dCACHE domain proteins can utilise both subdomains for ligand recognition.

## Introduction

*Helicobacter pylori* is a motile, gram-negative bacterium that infects over 50% of the world’s population^1^. *H*. *pylori* selectively colonises the gastric epithelium and is able to survive in the host stomach for years. Although the majority of the infected people remain asymptomatic, *H*. *pylori* infection can be associated with a range of gastroduodenal diseases, including gastritis, gastric and duodenal ulcers, and different types of cancer including mucosa-associated lymphoid tissue (MALT) lymphoma and gastric adenocarcinoma^2–4^. Directed motility, or chemotaxis, is important for the ability of *H*. *pylori* to swim through the highly acidic lumen towards the epithelium and to survive in the host environment under the conditions of constant turnover of the gastric mucosa. Non-motile or non-chemotactic mutants have been shown to be less effective in colonising the gastric mucosa and do not attain full infection compared to the wild type in animal models^5–8^.

**Figure 1:**
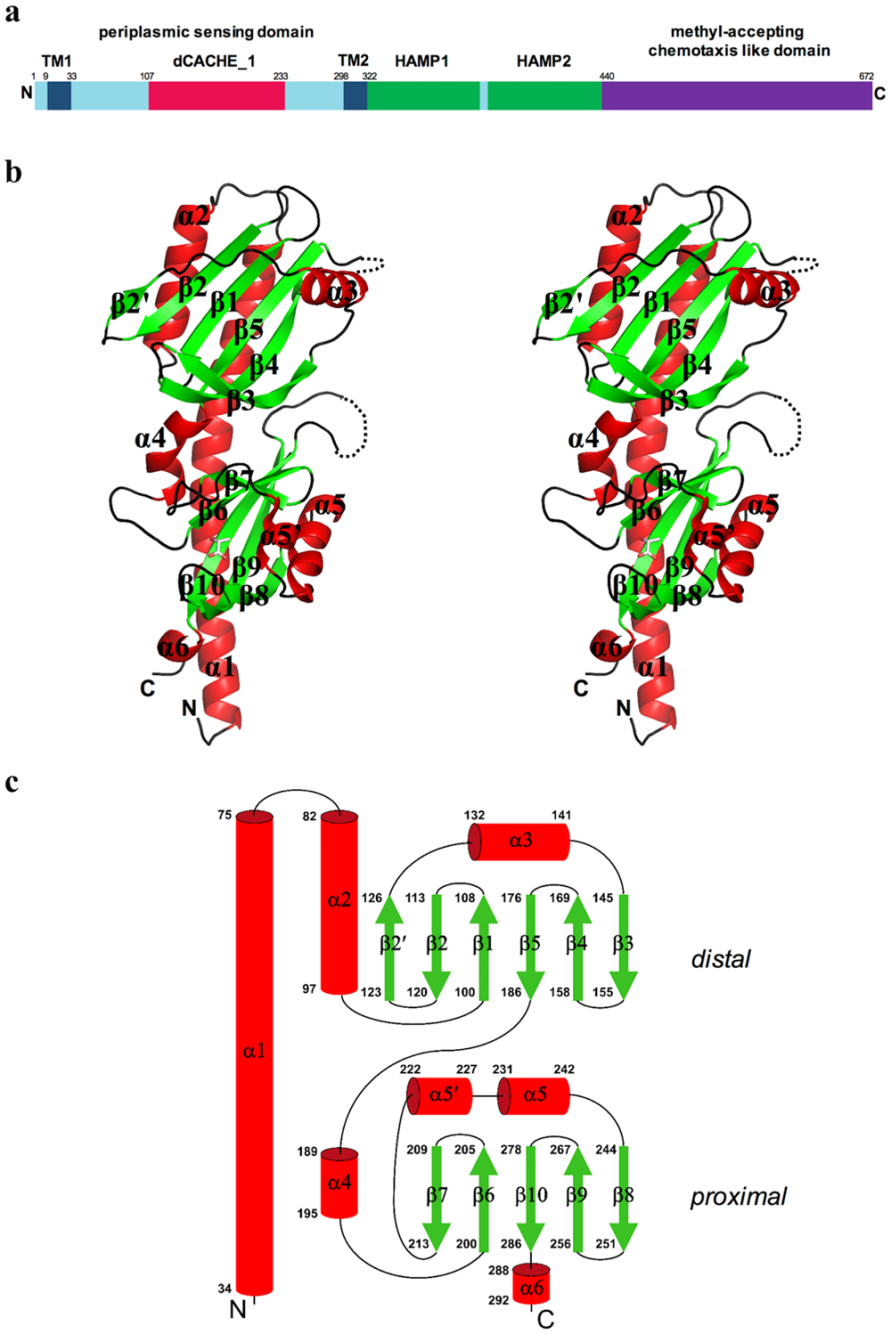
Overall fold of TlpC dCACHE LBD. (**a**) Domain organisation of TlpC, showing LBD location with respect to other structural elements. Transmembrane region (TM, dark blue); dCACHE_1 domain (red), HAMP domains (green); methyl-accepting chemotaxis-like domain (purple). (**b**) Stereo representation of structure of TlpC LBD monomer. (**c**) Topology of secondary structure elements of TlpC LBD. The α-helices are represented by rods and β-strands by arrows. The membrane-distal and membrane-proximal subdomains are labelled.

Chemotaxis allows motile bacteria to sense chemical cues and find optimal environments for growth by, for example, swimming towards favourable chemicals (chemoattractants) and away from harmful ones (repellents). Extracellular chemicals are sensed by chemoreceptors, also termed transducer-like proteins (Tlps). Most of the characterised Tlps are dimeric membrane proteins that comprise an extracytoplasmic ligand-binding domain (LBD), the transmembrane region, the HAMP (**h**istidine kinases, **a**denylyl cyclases, **m**ethyl-accepting protein, and **p**hosphatases) domain and the methyl-accepting (MA) domain (Fig. 1a), the latter transmitting information to a signalling cascade. The signal is relayed through the coupling protein CheW to the histidine protein kinase, CheA, which phosphorylates the response regulator protein, CheY, altering its affinity to the flagellar motors and, as a consequence, the direction (clockwise or counter-clockwise) in which they rotate^9^.

The chemotaxis pathway has been extensively studied in *Escherichia coli* ^10–12^. Recognition of cue molecules in this bacterium is mediated by five different chemoreceptors^9,12,13^. Four of them contain a periplasmic LBD with a 4-helix bundle (4HB) fold. The fifth receptor, Aer, has a cytosolic Per–Arnt–Sim (PAS) LBD and is involved in aerotaxis^14^.

Chemoreceptors have been classified according to the size of their LBD into cluster I (~150 amino acids) or cluster II (~250 amino acids)^15^. Much of what is known about bacterial chemoreceptors comes from studies on cluster I chemoreceptors with a 4HB LBD. However, more recent studies have shown that extra-cytoplasmic LBDs of chemoreceptors from different bacteria vary largely in their amino acid sequence and three-dimensional structure^16^ and, to date, additional structural families have been identified, including single CACHE (sCACHE)^17–19^, helical biomodular (HBM)^20,21^ and double CACHE (dCACHE) domains^19,22–25^.

In *H*. *pylori*, four chemoreceptors have been identified based on full genome sequence analysis: TlpA, TlpB, TlpC, and TlpD. TlpD is a soluble, cytoplasmic chemoreceptor that is involved in energy taxis^26^ and the repulsion response to reactive oxygen species^27^ and acid^28^. TlpA, TlpB, and TlpC are integral membrane proteins^29,30^. TlpA has been linked to recognition of bicarbonate and arginine as attractants^31^ and acid as a repellent^28^, whilst TlpB has been reported to detect acidic pH^17,30^ and the quorum-sensing molecule autoinducer-2 (AI-2)^32^ as repellents and direct the chemoattraction response to urea^33^. No signals have been associated with TlpC.

*H*. *pylori* additionally exhibits chemotactic responses to various other signals, including amino acids (aspartate, glutamate, asparagine, glutamine, histidine, proline, tyrosine, valine, leucine, serine and glycine)^34^, cholesterol^35^, bile acids (such as glycocholic, taurocholic, glycodeoxycholic, taurodeoxycholic, glycochenodeoxycholic and taurochenodeoxycholic acid)^34^, ZnCl_2_^36^ and NiCl_2_^31^. However, the recognition of these molecules has not been attributed to a specific chemoreceptor, and the mechanism by which these signals are sensed is currently unknown.

Amongst the four *H*. *pylori* chemoreceptors, only the periplasmic LBD of TlpB has been structurally characterised^17^. It is a homodimer of sCACHE modules^17,19^ – a feature that contrasts to the helical-bundle (4HB) modules of the extensively characterised aspartate receptor Tar from *Salmonella typhimurium* ^37^, the serine receptor Tsr from *E*. *coli* ^38^, and the McpS chemoreceptor from *Pseudomonas putida* ^20^. It is now recognised that the CACHE domain, either in its single sCACHE or double dCACHE form, is the most abundant extracellular sensing domain in prokaryotes, and is commonly found in two-component histidine kinases and chemoreceptors^16,19,22,23,39–41^.

TlpC is the least characterised chemoreceptor in *H*. *pylori*, and its natural ligand was unknown. *H*. *pylori tlpC* mutants are outcompeted by wild type during stomach colonisation, and TlpC modulates the chemotactic response to acid^29,36^. A BLAST search with the sequence of the sensing domain of TlpC against the structures deposited in the Protein Data Bank (PDB) identified no structural homologues of this domain. However, a pairwise comparison of profile Hidden Markov Models using the HHpred server^42^ predicted homology at the level of secondary structure to the sensing domains of family 1 histidine kinases (PDB entries 3lia, 3lib, 3lic, 3lid, 3lif)^43^ and chemoreceptors Tlp1 and Tlp3 from *Campylobacter jejuni* (PDB entries 4wy9 and 4xmr)^23,24^. These sensing modules belong to the recently redefined dCACHE_1 structural family^19^.

dCACHE domains consist of two structurally similar subdomains that each adopt a PAS-domain-like fold and are arranged in tandem, with one membrane- proximal and the other membrane-distal. dCACHE domain proteins can recognise their signal molecules directly or indirectly. Directly recognised ligands include amino acids^22,23,44–46^, pyrimidines^47^ and purines^48^. In all previously characterised dCACHE domains, direct sensing involves binding of the signal molecule to the membrane-distal, rather than membrane-proximal, subdomain, and no role for the membrane-proximal subdomain has been determined^22,23,43,44,49^.

In this paper, we report the crystal structure of LBD of *H*. *pylori* TlpC in complex with a small-molecule ligand that co-purified with the protein. The ligand was bound to the membrane-proximal subdomain. Based on the analysis of the electron density maps and the chemical nature of the ligand-binding pocket, we hypothesised and confirmed the ligand to be lactate. The location of the binding site has been validated by mutagenesis. We furthermore verified that lactate acts as an attractant for *H*. *pylori*, and that TlpC mediates the chemoattractant response. To the best of our knowledge, this is the first example of the dCACHE domain that directly recognises its ligand *via* the membrane-proximal module.

## Results

### Overall structure of TlpC LBD

The three-dimensional structure of recombinant *H*. *pylori* TlpC LBD (residues 34–297 plus six additional residues (GIDPFT) at the N-terminus, introduced as an artifact of the cloning procedure), was determined by X-ray crystallography using a single-wavelength anomalous dispersion (SAD) technique to a resolution of 2.2 Å. The TlpC LBD crystals (hereafter referred to as form A) belonged to the space group *C2*, with three molecules in the asymmetric unit related to each other by a three-fold pseudo-symmetry. The coordinates of these molecules were refined independently, and in the final model, they showed very similar backbone conformations that could be superimposed in a pairwise fashion with an overall root mean square deviation (r.m.s.d.) for the C_α_ atoms of 0.5–0.7 Å. Disordered regions 170–175, 271–274 and 295–297 were not seen in the electron density maps and could not be modelled.

In common with family 1 histidine kinases, the TlpC LBD has a dCACHE fold, and is composed of a membrane-proximal and membrane-distal PAS-like modules folding against the N-terminal and C-terminal halves of a long stalk helix, respectively (Fig. 1b). The TlpC LBD structure comprises six α-helices and 11 β-strands (Fig. 1c). The membrane-distal subdomain (residues 63–186) contains a six-stranded antiparallel β-sheet with the strand order 2′ 2 1 5 4 3, which is flanked on one side by an antiparallel two-helix bundle formed by helix α2 and the C-terminal half of helix α1, and on the other side by helix α3. The membrane-proximal subdomain (residues 34–62, 189–292) contains a five-stranded antiparallel β-sheet with the strand order 7 6 10 9 8. This β-sheet is flanked by an antiparallel two-helix bundle formed by helix α4 and the N-terminal half of helix α1 on one side, and by helices α5 and α5′ on the other side. Finally, an additional helix α6 forms an extension of strand β10 at the C-terminal end of TlpC LBD. The membrane-distal and membrane-proximal subdomains are intimately associated with each other, with a total buried surface area of 1169 Å^2^, which is equivalent to 16% of the total buried surface area (BSA) of an individual subdomain.

Analysis of the packing of monomers in the crystal lattice revealed head-to-tail arrangement of molecules, where the membrane-proximal subdomain of one subunit packs against the membrane-distal subdomain of the other, which is not likely to represent a physiologically relevant assembly. To determine the oligomeric state of TlpC LBD in solution, size-exclusion chromatography coupled to multi-angle light scattering (SEC-MALS) experiments were performed. TlpC LBD eluted as a single monodispersed peak in all conditions tested. The derived molecular weight of 28.8 kDa was close to the value calculated from the amino acid sequence of a monomer (30 kDa). Furthermore, the apparent hydrodynamic radius R_h_ of the particles in this peak (25 Å) was close the R_h_ value calculated from the crystal structure of a single TlpC LBD subunit (26 Å). In line with these results, analysis of the crystal packing using the PDBe PISA server (http://www.ebi.ac.uk/pdbe/pisa/) identified no quaternary structure and suggested that TlpC LBD is a monomer in the crystal.

### Comparison of TlpC LBD structure to other extracytoplasmic sensing domains

**Figure 2:**
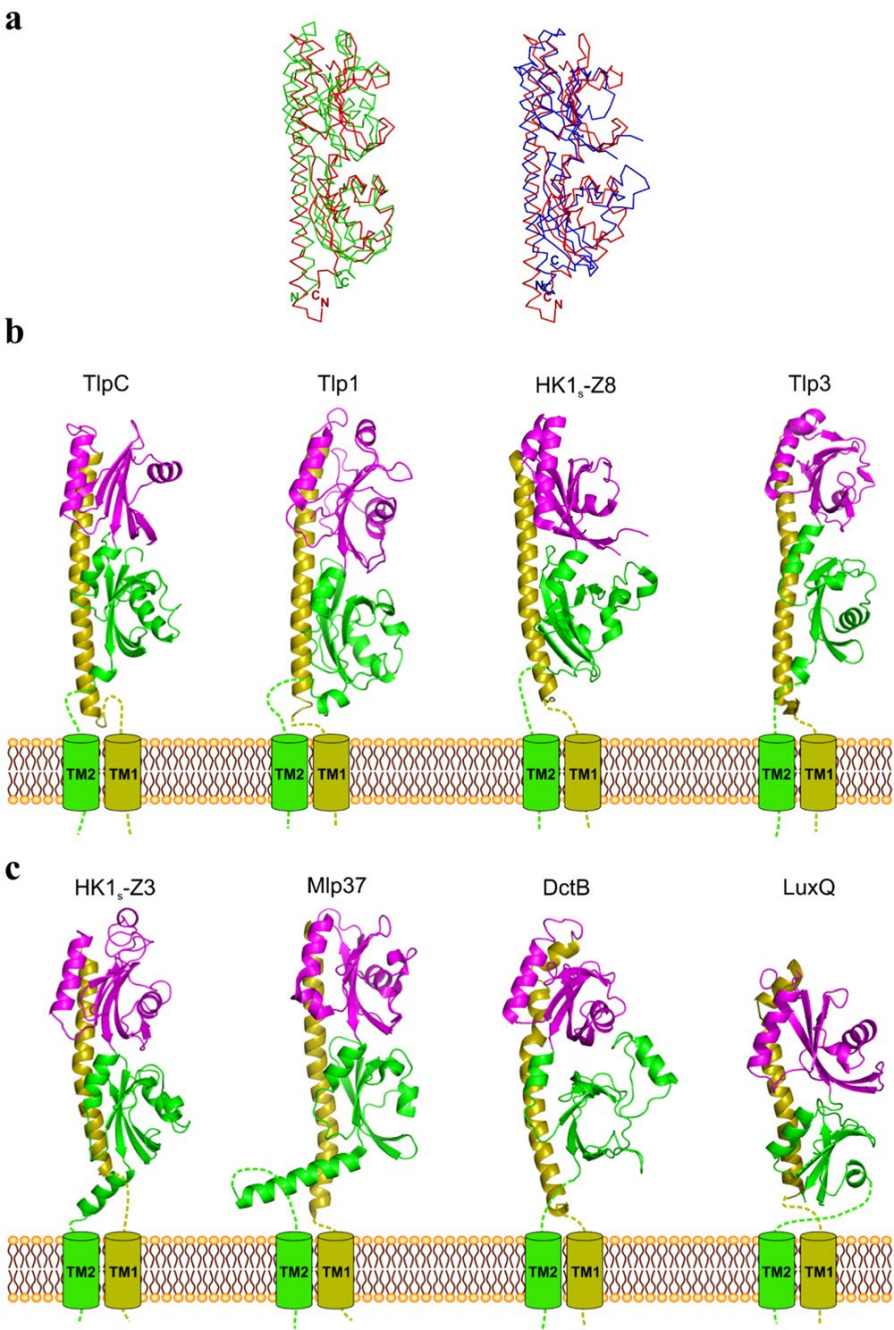
Comparison of dCACHE LBD of TlpC with structures of other dCACHE domains. (**a**,**b**) Structural superposition of TlpC LBD (C_α_ trace, in red) with dCACHE domains of (**a**) Tlp1 (C_α_, in green, PDB entry 4wy9^24^) and (**b**) HK1s-Z8 (C_α_, in blue, PDB entry 3lie^43^). (**c**) LBDs of TlpC, Tlp1 (PDB entry 4wy9^24^), HK1s-Z8 (PDB entry 3lie^43^), Tlp3 (PDB entry 4xmq^23^), HK1s-Z3 (PDB entry 3lib^43^), Mlp37 (PDB entry 5ave^51^), DctB (PDB entry 3by9^52^) and LuxQ (PDB entry 1zhh^53^).

In comparison of TlpC LBD atomic coordinates against structures deposited in the Protein Data Bank using PDBeFold^50^, the closest structural similarities were found with the dCACHE_1 sensory modules of chemoreceptors Tlp1 and Tlp3 from *C*. *jejuni* ^23,24^, and bacterial family 1 histidine kinases (HK) HK1_s_-Z8 (*Vibrio parahaemolyticus*), HK1_s_-Z3 and HK1_s_-Z2 (*Methanosarcina mazei*)^43^. TlpC LBD structure can be superimposed well over those of Tlp1 (Fig. 2a), HK1_s_-Z8 (Fig. 2b), HK1_s_-Z3, HK1_s_-Z2 and Tlp3 [root-mean-square deviation (r.m.s.d.) of 2.1, 2.2, 2.3, 2.5 and 2.9 Å for 285, 266, 271, 262 and 254 aligned C^α^ atoms from Tlp1, Z8, Z3, Z2 and Tlp3, respectively], despite the low overall sequence identity (<17%). The dCACHE fold adopted by the TlpC LBD has also been previously observed in sensing domains of chemoreceptor Mlp37^51^ and C4-dicarboxylate transport sensory HK DctB from *V*. *cholerae* ^52^ (Fig. 2c). Furthermore, this fold is remotely similar to the tandem-PAS fold of LBD of luminescence (lux) system HK LuxQ from *V*. *harveyi* ^53^ (Fig. 2c).

### Analysis of putative ligand-binding sites

**Figure 3:**
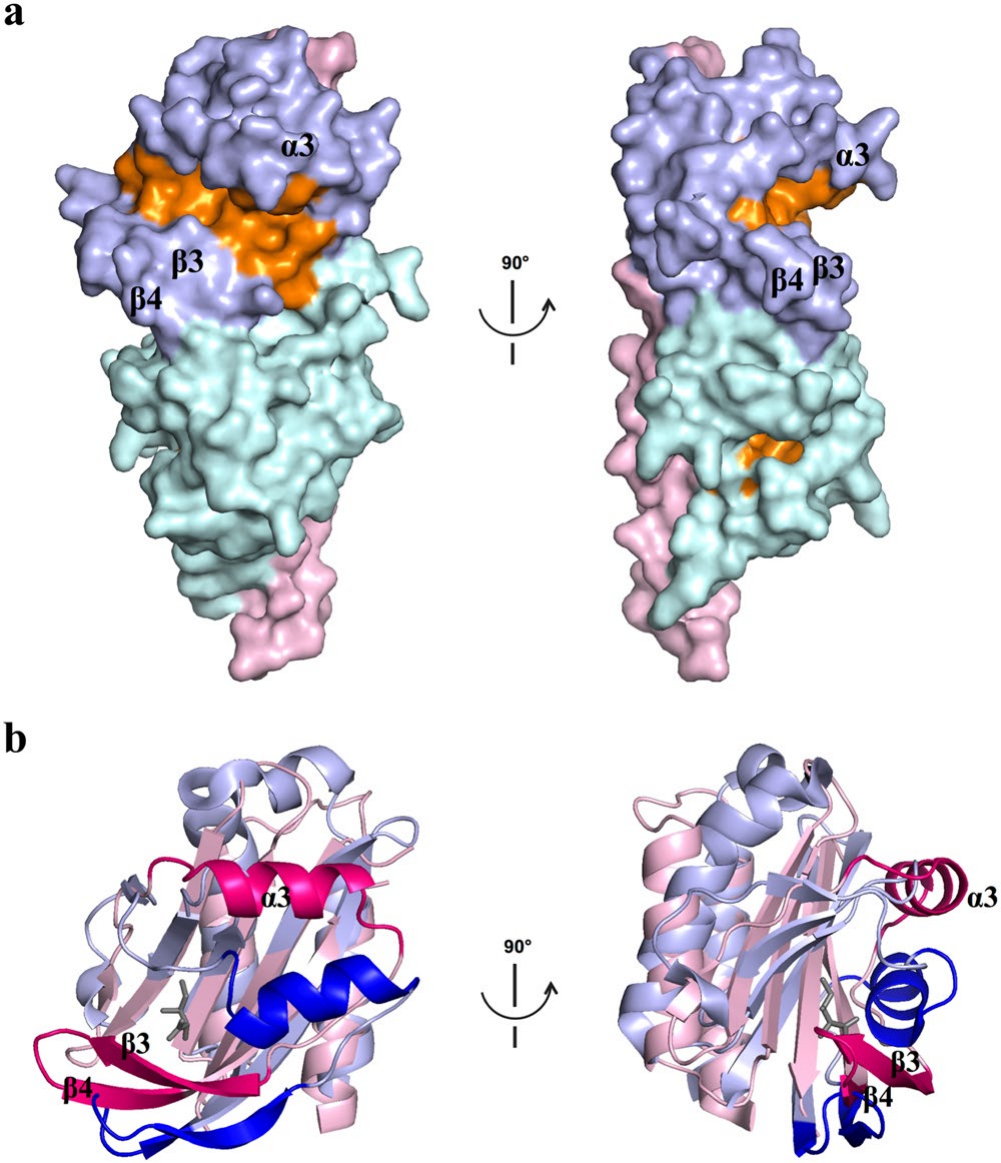
Putative ligand-binding sites of TlpC LBD. (**a**) Molecular surface of TlpC LBD with cavities and pockets coloured orange. The stalk helix is coloured pink, the membrane-distal module – light blue and the membrane-proximal module – cyan. (**b**) Structure superposition of membrane-distal modules of TlpC (pink) and Tlp3 (light blue) highlighting differences in position of helix α3 and β3-β4-tongue (coloured hot pink and blue in TlpC and Tlp3, respectively). Isoleucine bound to the membrane-distal module of Tlp3 is shown as grey sticks.

We next examined the putative ligand binding sites, starting with the membrane-distal module. Inspection of the structure of the membrane-distal subdomain around the region implicated in binding of small-molecule ligands in other dCACHE-containing proteins revealed a well-defined groove that runs along the full length of the β-sheet and is flanked on one side by helix α3 and the stretch of amino acid residues connecting β2′ and α3, and on the other side, by the β3-β4-tongue (Fig. 3a and b). This cleft is lined by mostly aliphatic and small hydrophilic residues and has the following approximate dimensions: 30 Å in length, 11 Å in width, and 9 Å in depth. Structural comparisons show that the groove in the membrane-distal domain of TlpC is significantly larger than the small pocket present in Tlp3 and other dCACHE sensing domains that recognise their small-molecule ligands directly^23,24^. Superimposition of the membrane-distal subdomains of TlpC and Tlp3 over 121 C^α^ atoms (r.m.s.d. of 2.9 Å) shows that helix α3 and the β3-β4-tongue in TlpC are positioned significantly further apart than the equivalent α-helix and β-tongue in Tlp3 (Fig. 3b). The cleft in TlpC appears too large to be a small-molecule-ligand binding site and could hypothetically fit a molecule of the size of a peptide, such as, for example, a loop or a terminal peptide of an-as-yet unidentified PBP.

**Figure 4:**
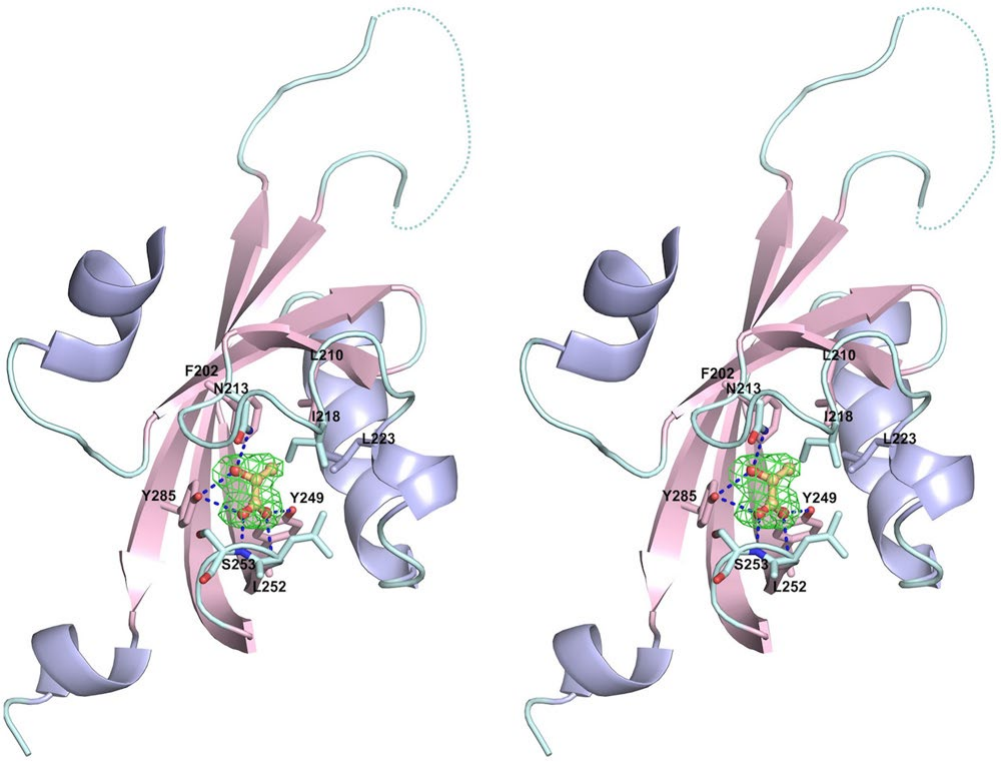
Architecture of ligand-binding site in membrane-proximal module of TlpC dCACHE domain. The (mFo - DFc) σ_A_-weighted electron density for lactate is shown in green. The map was calculated at 2.2 Å resolution and contoured at the 3.0 σ level. The lactate molecule is shown in all-atom ball-and-stick representation with C atoms coloured orange. The protein side chains that form direct contacts with lactate are shown in stick representation.

We then analysed the molecular surface of the membrane-proximal subdomain of TlpC LBD using CASTp^54^ with a probe radius of 1.4 Å. We detected a putative ligand-binding pocket with the surface area and solvent-accessible volume of 203 Å and 196 Å^3^, respectively (Fig. 3a). There was a clear electron density for a non-protein molecule bound in this pocket (Fig. 4). However, its shape did not match any of the components of the purification or crystallisation buffers, which suggested that the ligand trapped in the crystal could be a molecule that was present in the refolding mix or a product of proteolytic degradation of the sample.

### Identification of lactate as a ligand for TlpC

To identify the ligand captured by TlpC LBD, the protein was denatured to release the small molecules, and these were analysed by liquid chromatography-electrospray ionisation mass spectrometry (LC-ESI-MS). The negative ionisation mode MS data showed a small peak at m/z = 89.022 that was absent in the buffer control (Supplementary Fig. 1). Within the experimental error, this peak matched the chemical formula C_3_H_5_O_3_ (m/z 89.024). A search in the PubChem database (https://pubchem.ncbi.nlm.nih.gov/search/) identified 47 different compounds matching this formula. The shape of four of these (lactate, 1,1-dihydroxypropan-2-one, hydron-2-hydroxypropanoate and prop-2-ene-1,1,2-triol) matched the shape of the electron density in the membrane-proximal pocket. As lactate is the only one of these four compounds that is a natural metabolite produced during *E*. *coli* growth, we hypothesised that during refolding, the lactate was captured by the protein from the cell lysate.

**Figure 5:**
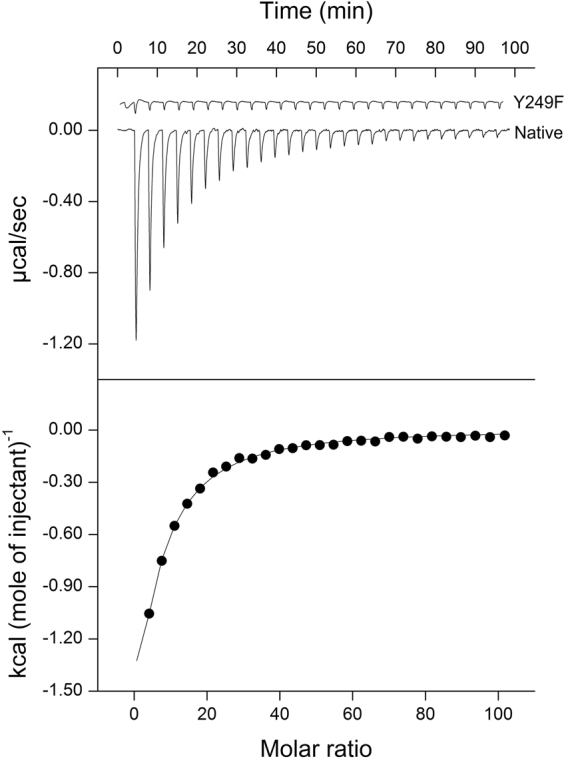
ITC titrations of TlpC LBD and its Y249F variant with lactate. The upper panel shows raw titration data, where each peak corresponds to the injection of 10 μl of 5 mM sodium *L-*lactate into a 1.45-ml reaction cell containing 10 μM protein. The lower panel shows the integrated and dilution-corrected peak areas of the titration plot.

Isothermal titration calorimetry (ITC) measurements confirmed that *L*-lactate binds exothermically to TlpC LBD with an apparent *K*_D_ of 155 μM ± 5 μM (Fig. 5). The binding is driven by a favourable enthalpy change (∆*H* = −20 kcal mol^−1^) and is associated with a minor unfavourable entropy change (T∆*S = −*1.29 kcal mol^−1^). This binding appears specific to lactate because no significant heat release or absorption was observed with pyruvate, malate or oxaloacetate, that are chemically similar and metabolically exchangeable with lactate (Supplementary Fig. 2).

### Validation of lactate binding site in membrane-proximal module of TlpC dCACHE domain

To establish whether lactate binds to the membrane-distal or membrane-proximal module, we determined the crystal structure of TlpC dCACHE domain co-crystallised with 10 mM *L*-lactate. The co-crystals with lactate were isomorphous to the form A crystals grown with no lactate in the crystallisation mix. Superposition of the protein contents of the two asymmetric units based on the overlap of 767 C_α_ atoms with an r.m.s.d. of 0.32 Å showed that, within the limit of the experimental error in the coordinates (0.33 Å for the co-crystal with lactate), their structures were essentially identical. Analysis of the electron density maps revealed no lactate binding sites other than the one in the membrane-proximal subdomain. This subdomain contained a lactate molecule bound in a very similar mode to that observed in the form A crystals grown with no added lactate (Fig. 4).

The lactate binding site is located in a pocket formed by residues F202, L210, N213, I218, L223, Y249, L252, S253, and Y285. Calculation of the accessible surface area (ASA) showed that lactate becomes almost completely shielded from the solvent upon binding to TlpC LBD, with 99.5% of its ASA buried by the protein. The carboxyl and hydroxyl groups of lactate form hydrogen bonds with the side chains of N213, Y249 and Y285, and with the main-chain amides of L252 and S253. The TlpC LBD/lactate complex is further stabilised by hydrophobic interactions between the methyl group of lactate and the side chains of F202, L210, I218 and L223 (Fig. 4).

**Figure 6:**
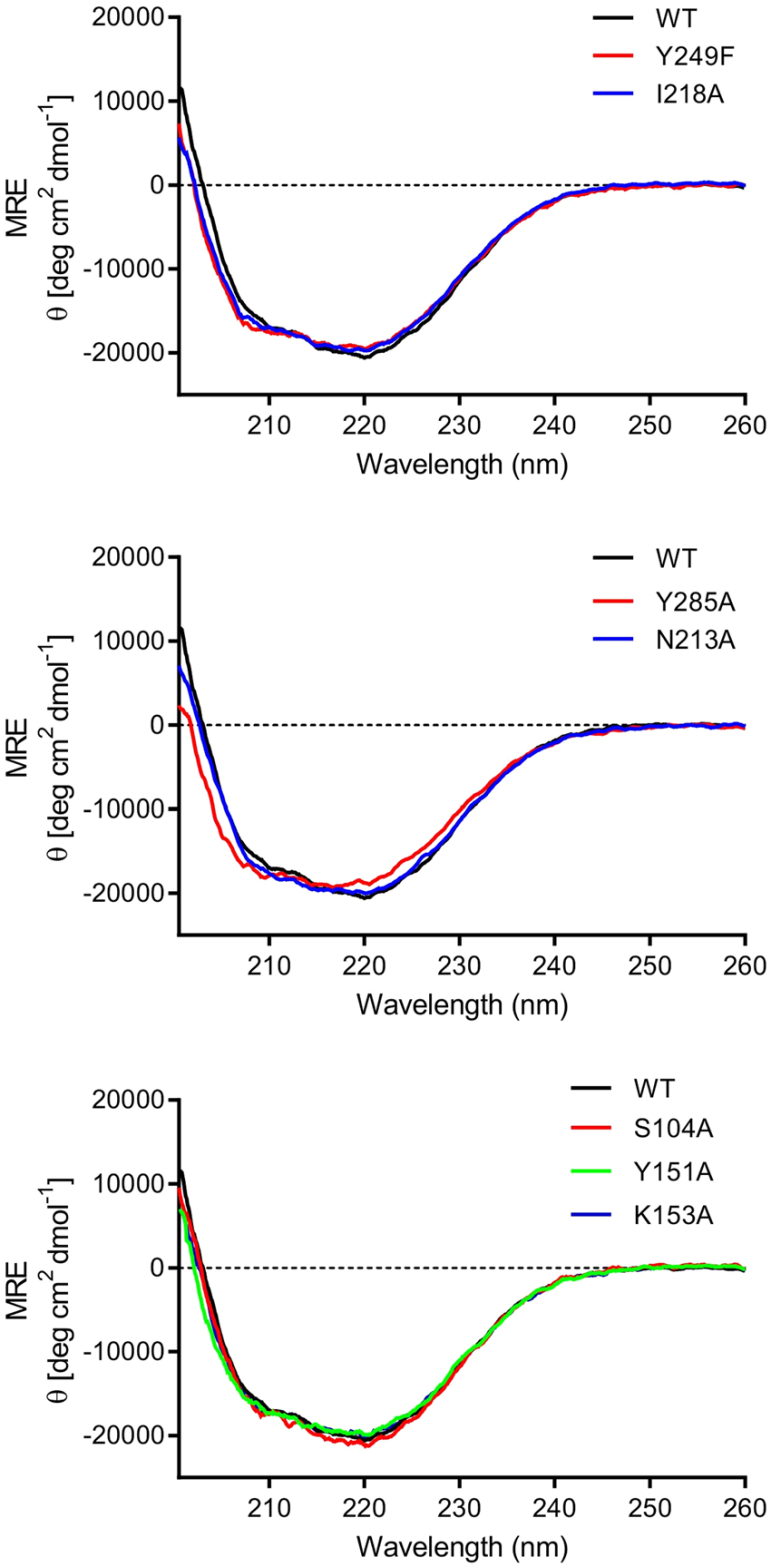
CD spectra of native TlpC LBD (WT) and its S104A, Y151A, K153A, N213A, I218A, Y285A and Y249F variants.

To evaluate the contribution of individual amino acids to the lactate binding, N213, I218 and Y285 were individually replaced with alanine and Y249 with phenylalanine, and the effect of the single-amino acid substitutions was assessed by isotitration calorimetry. Comparison of the circular dichroism spectra of the variants with that of native TlpC LBD showed no significant differences, indicating that the amino acid substitutions did not alter the secondary structure (Fig. 6). ITC measurements demonstrated that each of the N213A, I218A, Y285A and Y249F substitutions abolished the binding of lactate to TlpC LBD (Fig. 4 and Table 1). To further confirm that the membrane-distal subdomain does not bind lactate, the TlpC residues S104, Y151 and K153 – occupying the positions structurally equivalent to the ligand-binding residues Tyr118, Tyr167 and Thr170 in the membrane-distal subdomain of Tlp3 – were individually substituted with alanine. In contrast to the effect on the binding to the membrane-proximal domain, these substitutions only resulted in 2–3 fold reduction in the affinity to lactate, likely due to partial fold destabilisation rather than loss of interactions with the ligand.

**Table 1:** Thermodynamic parameters of lactate binding to TlpC LBD and its variants derived from ITC measurements.

Protein	*K*_D_	Enthalpy, ∆H (cal/mol)	Entropy, ∆S (cal/mol/degree)
TlpC LBD native	155.0 ± 5.0 µM	−21,323.3 ± 713.0	−54.1 ± 2.0
TlpC LBD N213A	>3,000	—	—
TlpC LBD I218A	3.1 ± 0.6 mM	−18,145.0 ± 49.0	−49.4 ± 0.2
TlpC LBD Y249A	>3,000	—	—
TlpC LBD Y285F	>3,000	—	—
TlpC LBD F202A	>3,000	—	—
TlpC LBD K223A	>3,000	—	—
TlpC LBD S104A	359.0 ± 3.0 µM	−13,105 ± 318.0	−28.2 ± 1.0
TlpC LBD Y151A	278.5 ± 2.0 µM	−12,040 ± 250.2	−24.1 ± 1.0
TlpC LBD K153A	467.2 ± 5.0 µM	−12,730 ± 345.0	−27.5 ± 1.0

### *H*. *pylori* TlpC mediates positive chemotactic response to lactate

**Figure 7:**
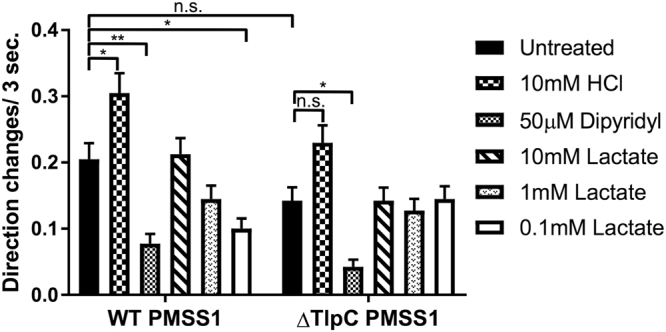
Lactate triggers a TlpC-dependent attractant response in *H*. *pylori*. Cultures of isogenic wild-type (WT) and Δ*tlpC* (∆TlpC) *H*. *pylori* PMSS1 were grown in BB10 overnight, treated with various concentrations of sodium *L-*lactate or control compounds as indicated, and then immediately filmed. Direction changes were enumerated over a 3 second period in at least 200 cells per treatment in two biological replicates. 10 mM HCl or 50 μM dipyridyl serve as known repellent and attractant response controls, respectively. Error bars represent the standard error of the mean. *p < 0.05; **p < 0.01, comparisons performed using a two-way ANOVA, followed by Tukey’s pairwise comparisons (α = 0.05). There were no significant differences in the basal behaviour between wild type and its *tlpC* mutant.

To test the physiological relevance of the observed specific interaction between TlpC and lactate, we assessed the lactate chemotactic response of *H*. *pylori*. Although there are isolates of the laboratory *H*. *pylori* strain 26695 that are motile^55^ this strain is prone to motility loss and difficult to use in motility evaluation. We therefore used the human isolate pre-mouse SS1 (PMSS1)^56^, which displays a high level of reliable motility, and has been studied for chemotaxis responses in recent publications^28^. The TlpC ligand-binding domain from 26695 and PMSS1 are identical (Supplementary Fig. 3), so we reasoned both proteins would respond similarly to lactate. We assessed whether lactate is an *H*. *pylori* attractant or repellent using a swimming assay that enumerates flagellar-based bacterial reversals, a common read out for a chemotactic response^57^. In this assay, attractants cause decreased and repellents cause increased direction changes^26,27,32^. Wild-type *H*. *pylori* showed a significant response to 0.1 mM lactate in this assay, but lost the response at higher concentrations (Fig. 7).

To account for possible chemotactic effects due to pH change upon sodium *L*-lactate treatment of BB10, the pH of the media with and without treatment was assessed. While treatment with 10 mM HCl decreased the pH by more than 1.5 pH units, treatment with any concentration of sodium *L*-lactate only decreased the BB10 pH by less than 0.05 pH units. Furthermore, the pH difference between the highest and lowest amount of sodium *L*-lactate (10 mM and 0.1 mM) was only ~0.01. This analysis suggests that sodium *L*-lactate did not substantially change the medium pH, and thus any effect due to pH change upon sodium *L*-lactate treatment were likely negligible compared to chemotactic effects due to sodium *L*-lactate itself.

We next examined whether TlpC was required for this lactate chemotaxis response. We generated an isogenic null mutant strain lacking *tlpC* (∆*tlpC*). *tlpC* is part of a single gene operon^58,59^, and thus mutations are unlikely to have polar effects. Consistent with this idea of specificity, the ∆*tlpC* mutant retained chemotaxis responses to signals that act through other chemoreceptors: the known repellent HCl that acts through TlpB, TlpA, and TlpD, as well as the attractant dipyridyl that acts through TlpD^27,28,30^ (Fig. 7). These responses confirmed that the ∆*tlpC* mutant was not generally chemotaxis defective. Deletion of *tlpC*, however, abolished the chemotactic response to lactate at all tested concentrations (Fig. 7). Additionally, while ∆*tlpC* PMSS1 *H*. *pylori* displayed fewer reversals on average compared to WT PMSS1, this difference in basal reversal frequency was not significant (Fig. 7). This data thus supports that TlpC is the chemoreceptor for lactate in *H*. *pylori*.

## Discussion

dCACHE domains of bacterial chemoreceptors and histidine kinases consist of two subdomains, membrane-distal and membrane-proximal, each of which could, in principle, harbour a binding site for a small signal or regulatory molecule. In all previously characterised dCACHE domains, including LBDs of Tlp3 from *C*. *jejuni* ^23^, Mlp37 from *V*. *paraehemoliticus* ^51^, McpB and McpC from *B*. *subtilis* ^22,45^, and Mlp24 from *V*. *cholerae* ^60^, direct sensing involved binding of the signal molecule to the membrane-distal subdomain. Our analysis of the structural basis of lactate recognition by *H*. *pylori* chemoreceptor TlpC changes this paradigm regarding the mechanism of sensing by dCACHE domain by providing the first example where direct sensing of the signal molecule is mediated by the ligand binding to the membrane-proximal, rather than membrane-distal, subdomain.

This result has important implications for the conceptual framework of dCACHE-mediated sensing and signal transmission across the membrane. As this and previous studies showed, the membrane-distal and membrane-proximal modules of dCACHE are intimately associated with each other, and are therefore structurally and dynamically coupled^23,24^. For example, we previously demonstrated using X-ray crystallography that, upon binding to an attractant, the dCACHE membrane-distal subdomain of *C*. *jejuni* Tlp3 closes around the ligand and loses its tight association with the membrane-proximal domain, which, as a result, adopts a more open conformation^23^. The structural coupling of the membrane-distal and membrane-proximal subdomains is consistent with the finding that signalling across the membrane presumably can be triggered by direct ligand binding to either subdomain – the membrane-proximal subdomain, as in TlpC, or membrane-distal subdomain as in Tlp3, Mlp37, McpB or McpC. Furthermore, one cannot eliminate the possibility that different ligands may signal through the same receptor, with some binding to the membrane-distal and others to the membrane-proximal subdomain. Indeed, all membrane-proximal subdomains of dCACHE sensing domains characterised to date contain a putative small ligand-binding pocket, including those that sense ligands with the membrane-distal domain^23,24,43,51^. For example, the structural study of LBD of *C*. *jejuni* chemoreceptor Tlp1 revealed an acetate ion bound to the dCACHE membrane-proximal module^24^. Acetate has not yet been found to trigger a chemotaxis response in *C*. *jejuni* ^61^. However, it has been shown to induce either a positive or negative chemotactic response in other species^18,62–64^.

Apart from implicating the membrane-proximal, rather than membrane-distal, subdomain in direct sensing of a small molecule ligand, our crystallographic analysis revealed one more difference between the dCACHE domain of TlpC and that of other structurally characterised chemoreceptors of this type – the presence of a long groove in the membrane-distal subdomain, instead of a small well-defined pocket^23,43,51^. This groove might represent a putative binding site for a larger molecule, such as an intermediate ligand-binding protein from the periplasmic binding protein (PBP) family. PBP-mediated sensing is used for chemotaxis in other bacteria, where some PBPs have a function of a primary chemoreceptor that recognises and binds a small molecule in the periplasm, and, in the ligand-bound form, associates with its cognate, membrane-bound transducer-like protein, initiating the signal^65^. There are at least six putative PBPs encoded in the *H*. *pylori* genome which have been shown to bind autoinducer 2^66^ and nickel^67^, and proposed to bind other compounds including peptides^68^, molybdenum, amino acids, and iron^69^. Whilst the membrane-proximal domain of TlpC mediates direct sensing of lactate, its membrane-distal domain – the shape of which does not imply small-molecule binding – may partner with a PBP to sense some other ligand.

Our analysis of the chemotactic behaviour of wild-type *H*. *pylori* showed that lactate induced an attractant response in a concentration-dependent manner, and that this response was drastically reduced in a chemotactically competent isogenic Δ*tlpC* mutant, demonstrating that TlpC is the primary chemoreceptor for lactate in *H*. *pylori*. Within the tested range of concentrations of lactate, the response was strongest at 0.1 mM, detectable at 1.0 mM, and not detectable at 10 mM. Putting the observed TlpC-dependent chemotactic behaviour towards *L*-lactate in the context of the receptor-ligand interactions, we note that the order of the optimal concentration at which lactate is sensed by *H*. *pylori* as attractant (0.1 mM) is the same as the order of the dissociation constant *K*_D_ (0.155 mM) for its binding to the TlpC dCACHE domain. *L*-lactate, secreted by gastric mucous cells, reaches the concentration of 0.3–1 mM in gastric juice^70,71^. Presumably lactate forms a gradient with its highest amount at the source, the cells, but the stomach distribution of lactate is not known. Lactate is known to promote *H*. *pylori* growth in the stomach^72^ and in media that is lacking dextrose, suggesting it can serve as either a carbon or energy source, or both^73^. Metabolically, lactate can be generated by lactate dehydrogenase (LDH) from pyruvic acid as the end product of glycolysis. However, LDH can also catalyse the reverse reaction, converting lactate into pyruvate^74,75^. Thus, if exogenous lactate was imported into *H*. *pylori*, it could be oxidised into pyruvate, which would then enter the tricarboxylic acid cycle. Alternatively, lactate can donate electrons to NADH and, in turn, to the electron transport chain to enhance proton motive force and bacterial energy levels^76^. In *H*. *pylori*, the proteins necessary for the import and utilisation of lactate have been identified^69,73^, including two lactate permeases and two LDHs^73^. At least one of the lactate utilisation genes has been shown to be required for stomach colonisation, supporting the importance of this process *in vivo* ^77^.

TlpC mutants have mouse stomach colonisation defects but only when competing with wild type^29^. This phenotype is consistent with the idea that either lactate is limiting and wild type utilises it more efficiently, or that wild type follows a lactate gradient and occupies key niches before the *tlpC* mutant can get there. Thus, the *in vivo* fitness defect observed with isogenic TlpC mutants is consistent with an inability to efficiently access and catabolise lactate *in vivo* ^29^.

Interestingly, several *H*. *pylori* lab strains appear to lack TlpC protein expression^27^. One of these strains, G27, has a single base indel that creates a frameshift and results in loss of TlpC expression^27^. Another, B128, also has a *tlpC* frameshift but its gerbil-selected daughter strain, 7.13, has regained TlpC expression^27^. These findings suggest that TlpC is not required for lab growth. The stomach may provide different selective pressures such that TlpC expression is an advantage. In support of this idea, four clinical *H*. *pylori* isolates analysed all expressed TlpC^27^.

The dissociation constant for lactate binding to the TlpC dCACHE domain falls within the middle of the range of values reported for ligand binding by other CACHE domains (*e*.*g*. 23–356 µM for *Pseudomonas syringae* pv. *actinidiae* PscD LBD^78^, 0.6–373 µM for *P*. *putida* KT2440R McpA LBD^79^, 1–1000 µM for *B*. *subtilis* McpC LBD^22^). Lactate is also a chemoattractant for *Pseudomonas aeruginosa* ^18^. *P*. *aeruginosa* senses lactate *via* an sCACHE domain receptor named McpP. While this receptor has not yet been crystallised, it is known to bind lactate with similar affinity to TlpC, with a *K*_D_ of 107 µM^18^. McpP additionally binds acetate, pyruvate, and propionate. McpP is similar to several other sCACHE chemoreceptors, suggesting chemotaxis toward lactate and related C2 and C3 carboxylic acids may be widespread.

Our observation that a higher concentration of lactate (10 mM) did not elicit a positive chemotactic response in *H*. *pylori* is in agreement with the reports that, at a concentration of 10 mM or above, lactate has an inhibitory effect on *H*. *pylori* growth^72,80^. The anti-*H*. *pylori* activity of high levels of exogenous lactate was first observed in co-cultures with lactic acid bacteria (LAB)^81–83^. Lin *et al*.^84^ demonstrated that short chain fatty acids (SCFA) (acetic, formic, propionic, butyric and lactic acid) secreted by LAB, and the associated low pH values reduce *H*. *pylori* viability, with lactic acid exhibiting the strongest inhibitory effect out of all tested SCFA^84,85^. Although the exact mechanism by which high levels of lactic acid exert anti-*H*. *pylori* activity remains unclear, it is likely a combination of its inhibitory effect on *H*. *pylori* urease activity and the reduced ability of *H*. *pylori* to survive at low pH in the absence of urea^80,84–87^.

Full-length chemoreceptors function as trimers of dimers^88^. Although the degree and mechanism of the contribution dCACHE LBDs make to oligomerisation *in vivo* remains to be established, previous crystallographic studies on the dCACHE domains of *C*. *jejuni* Tlp3^23^, *M*. *mazei* HK1_s_-Z3^43^ and *V*. *cholerae* DctB^52^ suggested that they likely dimerise through their stalk helix, with the twofold axis approximately perpendicular to the membrane plane. The dimerisation forces between isolated dCACHE domains are weak, as all domains of this type characterised so far, including that of *H*. *pylori* TlpC (this study), Tlp1 and Tlp3 from *C*. *jejuni* ^23,24^, CtaA and CtaB from *Pseudomonas fluorescens* ^89,90^, VfcA from *Vibrio fischeri* ^91^, and PctA from *P*. *putida* ^92^, are monomeric in solution. Our analysis showed that the isolated recombinant dCACHE LBD of TlpC is monomeric in the crystal as well. However, the observed structural similarity between LBDs of TlpC, Tlp3, HK1_s_-Z3 and DctB allows for the possibility that, in the context of the membrane-embedded full-length receptor, TlpC LBD may also dimerise through its stalk helix.

In conclusion, this study reports the first example of the dCACHE type chemoreceptor that directly senses its ligand *via* its membrane-proximal subdomain, and that *H*. *pylori* seeks out lactate using chemotaxis. It adds to the mounting evidence that dCACHE sensing domains have evolved to recognise their ligands *via* several different direct and indirect mechanisms that may utilise either the membrane-distal, or the membrane-proximal, subdomain, or both. This raises an intriguing question about whether, despite this diversity, different dCACHE sensing domains share a common mechanism of signal transduction across the membrane.

## Methods

### Site-directed mutagenesis, protein expression, and purification

The expression vectors for single-point variants of TlpC LBD in which S104, Y151, K153, N213, I218 or Y285 were replaced by alanine, and Y249 by phenylalanine, were prepared from a TlpC-expressing plasmid described previously^93^. This plasmid expresses codon-optimised *H*. *pylori* 26695 TlpC LBD consisting of amino acid residues 34–297 (Fig. 1a). Mutants were created using the QuikChange Mutagenesis Kit (Stratagene). TlpC LBD and its variants were expressed and purified following the previously published procedure^93^.

### Crystallisation, data collection and structure determination

Form A crystals of TlpC LBD were obtained as described^93^. The crystals grew in space group *C*2 (Table 2) and contained three monomers in the asymmetric unit. Co-crystallisation with 10 mM sodium *L*-lactate under similar conditions produced the crystals of the TlpC LBD/lactate complex that were isomorphous with form A crystals (Table 2). Two platinum derivatives were obtained by soaking the TlpC LBD crystals in either potassium tetrachloroplatinate (1 mM) or potassium hexachloroplatinate (1 mM). The derivative crystals belonged to space group *P*321 (form B) with a monomer in the asymmetric unit (Table 2). Native X-ray diffraction data (λ = 0.95 Å) and SAD data for the derivatives (λ = 1.07 Å) were collected on the MX1 and MX2 beamlines of the Australian Synchrotron (AS)^94^. All data were processed with *iMOSFLM* ^95^ and scaled with AIMLESS^96^ from the CCP4 software suite^97^ (Table 2).

**Table 2:** X-ray Data collection and processing statistics. Values in parentheses are for the highest resolution shell.

Dataset	Native	K_2_PtCl_4_	K_2_PtCl_6_	Co-crystal with 10 mM lactate
Space group	*C*2	*P*321	*P*321	*C*2
*a*, *b*, *c* (Å)	189.3, 103.2, 61.8	102.7, 102.7, 62.4	102.5, 102.5, 63.0	188.5, 102.6, 61.2
β (°)	98.3			98.5
Resolution range (Å)	30.6–2.2	62.4–3.3	51.4–3.3	30.0–2.5
	(2.31–2.20)	(3.48–3.30)	(3.48–3.30)	(2.60–2.50)
R_merge_	0.062 (0.280)	0.095 (0.364)	0.096 (0.302)	0.069 (0.341)
Average I/σ(I)	13.3	18.2	18.1	58.5
Completeness (%)	98 (96)	99.9 (99.9)	99.9 (99.9)	99.8 (99.9)
Redundancy	3.6	10.5	10.5	3.6
Anomalous redundancy		5.5	5.4	
Observed reflections	215,101	62,341	62,541	505,592
Unique reflections	59,028	5,941	5,975	40,012

The two isomorphous SAD data sets were used to locate the platinum sites and calculate the phases for the form B crystals with Autosol^98^ from the PHENIX software suite^99^. The resulting phase set (overall figure of merit of 0.30 for data between 51.4 and 3.3 Å) was used to generate an initial partial model for a TlpC LBD monomer with AutoBuild (PHENIX). This model was used for phasing the form A data for the native crystal and the co-crystal with lactate by molecular replacement. Refinement statistics and stereochemistry are given in Table 3. For both models, all the non-glycine residues lie in permitted regions of the Ramachandran plot, with 97% of these in the most favoured regions.

**Table 3:** Refinement statistics.

Data set	TlpC native	Co-crystal with 10 mM lactate
Resolution range (Å)	30.6–2.2	30.0–2.5
No. reflections	59,028	40,012
R_work_/R_free_^a^	0.182/0.218	0.192/0.251
No. atoms		
Protein	6310	6212
Water	452	66
Lactate	18	18
*B*-factors		
Average *B* (protein atoms) (Å^2^)	46	42
Average *B* (water molecules) (Å^2^)	52	37
Average *B* (lactate) (Å^2^)	41	41
R.m.s. deviations from ideality		
Bond lengths (Å)	0.017	0.008
Bond angles (°)	1.4	1.1

^a^The R_free_ was calculated on 5% of the data omitted at random.

### SEC-MALS analysis

The hydrated molecular mass and hydrodynamic radius of TlpC LBD in solution were determined by SEC-MALS. Protein was dialysed against buffer *A* containing 100 mM Tris-HCl pH 8.0 and 150 mM NaCl, and concentrated to 3 mg ml^−1^. A 100 µl sample was loaded onto a WTC-030S5 SEC column (Wyatt Technology Corporation) pre-equilibrated with the same buffer flowing at 0.4 ml min^−1^. The eluate was passed through an inline DAWN HELEOS light scattering detector, an Optilab T-rEX differential refractive index detector and a quasi-elastic light scattering detector (WyattQELS, Wyatt Technology Corporation). The experiment was repeated in the presence of 10 mM sodium *L-*lactate. Calculations of the molecular mass and hydrodynamic radius from the intensity of the scattered light and refractive index were performed using ASTRA 6.0 (Wyatt) (Table 4). Theoretical calculations of the hydrodynamic radius from the crystal structure were carried out using HYDROPRO version 10^100^.

**Table 4:** Dynamic light-scattering results.

Sample	Polydispersity	Molecular weight (kDa)	*R*_h_ (nm)
TlpC LBD	1.0	28.8	2.5
TlpC LBD + sodium *L-*lactate	1.0	27	2.6
BSA	1.0	63.9	3.6

### LC-ESI-MS analysis

Identification of the ligand captured by TlpC LBD was achieved by extracting small molecules from the purified protein and measuring their masses by LC-ESI-MS. TlpC LBD (30 µM in buffer *A*) was unfolded by boiling at 100 °C for 15 min and then pelleted by centrifugation. Buffer *A* subjected to the same procedure was used as a negative control. 200 µl of the supernatant was directly infused into MicrOTOF-Q quadrupole time-of-flight (TOF) mass spectrometer (Bruker Daltonics), and then nebulised and ionised using the Bruker electrospray source. Data were acquired in both positive and negative ion ESI modes over the mass range of 70 to 200 Daltons. The spectra were processed using the Data Analysis software version 3.4 (Bruker Daltonics).

### CD analysis

CD spectroscopy was used to compare secondary structure composition of TlpC LBD and its single-point variants. Protein samples (0.1 mg ml^−1^) were dialysed against buffer *B* (10 mM sodium phosphate pH 7.4, 150 mM NaCl). Far-UV CD spectra were recorded over the wavelength range 200–260 nm at 25 °C with the scan rate of 20 nm min^−1^ using a JASCO J-815 spectropolarimeter. The spectra were measured in triplicate, averaged and smoothed using the Savitzky-Golay algorithm with a radius of 25^101^. Raw data were converted to mean residue ellipticity θ (in deg cm^2^ dmol^−1^)^102^.

### ITC experiments

TlpC LBD and its variants were dialysed against buffer *A*. 5 mM solutions of sodium lactate, sodium pyruvate, sodium malate and sodium oxaloacetate were prepared by dissolving them in the dialysis buffer. Measurements were performed on a MicroCal VP-ITC instrument microcalorimeter (MicroCal) at 25 °C. Protein (10 µM) in a 1.45-ml sample cell was injected with 25 successive 10-μl aliquots of the lactate solution. Binding isotherms were generated by plotting the heat change evolved per injection versus molar ratio of lactate to protein. The data was fitted to a single-site binding model using non-linear least-squares regression (Origin 7, OriginLab, USA), yielding the binding enthalpy ∆H, dissociation constant *K*_D,_ and binding entropy ∆H. Each experiment was repeated three times.

### Construction of isogenic Δ*tlpC* mutant in *H*. *pylori* strain PMSS1

The PMSS1 Δ*tlpC* mutant was created by natural transformation of wild-type PMSS1 with 5 μg of ∆*tlpC::cat* SS1 genomic DNA^29,56^. Chloramphenicol-resistant mutants were selected using 10 μg/ml chloramphenicol on Columbia Horse Blood Agar as previously described^29^. Mutation of *tlpC* was confirmed by PCR and western blot.

### Chemotaxis assay

Swimming behaviour assays were done with *H*. *pylori* PMSS1 strains described above grown in Brucella broth (BD BBL/Fisher) with 10% FBS (Life Technologies) (BB10), with shaking, at 37 °C, under microaerobic conditions of 5% O_2_, 10% CO_2_, balance N_2_. Overnight cultures (~OD_600_ 0.25–0.5) were diluted to an OD_600_ of 0.1 in fresh BB10, and then incubated as above until an OD_600_ of 0.12–0.15 was reached. Motile, OD_600_ 0.12–0.15, cultures were treated with sodium *L*-lactate (0.1 mM, 1 mM, 10 mM) or an equal volume of H_2_O as an untreated control. As a repellent control, 10 mM HCL was used as done previously^30,32,36^. As an attractant control, 50 μM dipyridyl was used as done previously^27^. Dipyridyl results in fewer directions changes, an attractant response, dependent on chemotaxis in general and TlpD specifically. Dipyridyl induces and attractant response as it counters reactive oxygen species *via* chelation of iron^27^. The pH of BB10 upon treatment was independently assessed using a Denver Instruments pH meter. Cultures were filmed immediately after ligand addition at 400x magnification using a Hamamatsu Digital Camera C4742-95 with the μManager software (Version 1.4.22), mounted on a Nikon Eclipse E600 phase contrast microscope^103^ (Supplementary videos 1–12). Videos were relabeled to blind the observer to the strain identity. For each sample, >100 3-s-long bacterial tracks from two independent cultures were analysed manually to identify stops followed by direction changes and to calculate the average number of direction changes in 3 s. Statistical analysis of the data for treated versus untreated samples was performed using a Student’s t-test.

### PDB submission codes

The atomic coordinates and structure factors of the TlpC LBD/lactate complex obtained at 2.2 Å resolution have been deposited in the Protein Data Bank (http://www.rcsb.org) under accession code 5wbf.

## Electronic supplementary material

Supplementary Information

Supplementary video 1

Supplementary video 2

Supplementary video 3

Supplementary Video 4

Supplementary video 5

Supplementary video 6

Supplementary video 7

Supplementary video 8

Supplementary video 9

Supplementary video 10

Supplementary video 11

Supplementary video 12
