# Supplementary material for: Helicobacter pylori chemoreceptor TlpC mediates chemotaxis to lactate
Source: Sci Rep. 2017 Oct 26;7:14089. doi: 10.1038/s41598-017-14372-2 (PMC5658362; doi:10.1038/s41598-017-14372-2)
Supplement: Supplementary file 1 — Supplementary Information [file 41598_2017_14372_MOESM1_ESM.pdf]

Supplementary Information

Helicobacter pylori chemoreceptor TlpC mediates chemotaxis to lactate

Mayra A. Machuca, Kevin S. Johnson, Yu C. Liu, David L. Steer, Karen M. Ottemann, Anna Roujeinikova

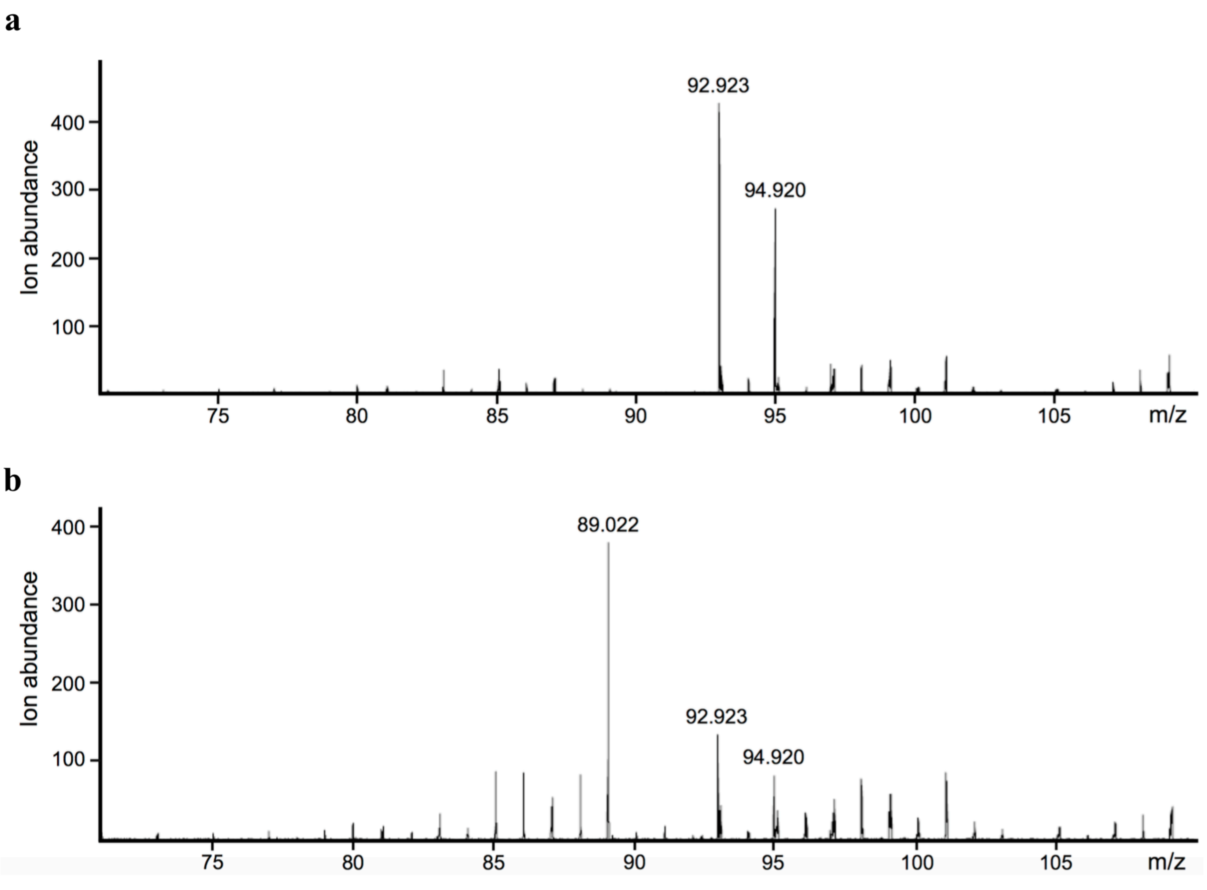

Supplementary Figure 1. Negative-ion-mode ESI-MS chromatogram. (**a**) Buffer control, (**b**) TlpC LBD in same buffer.

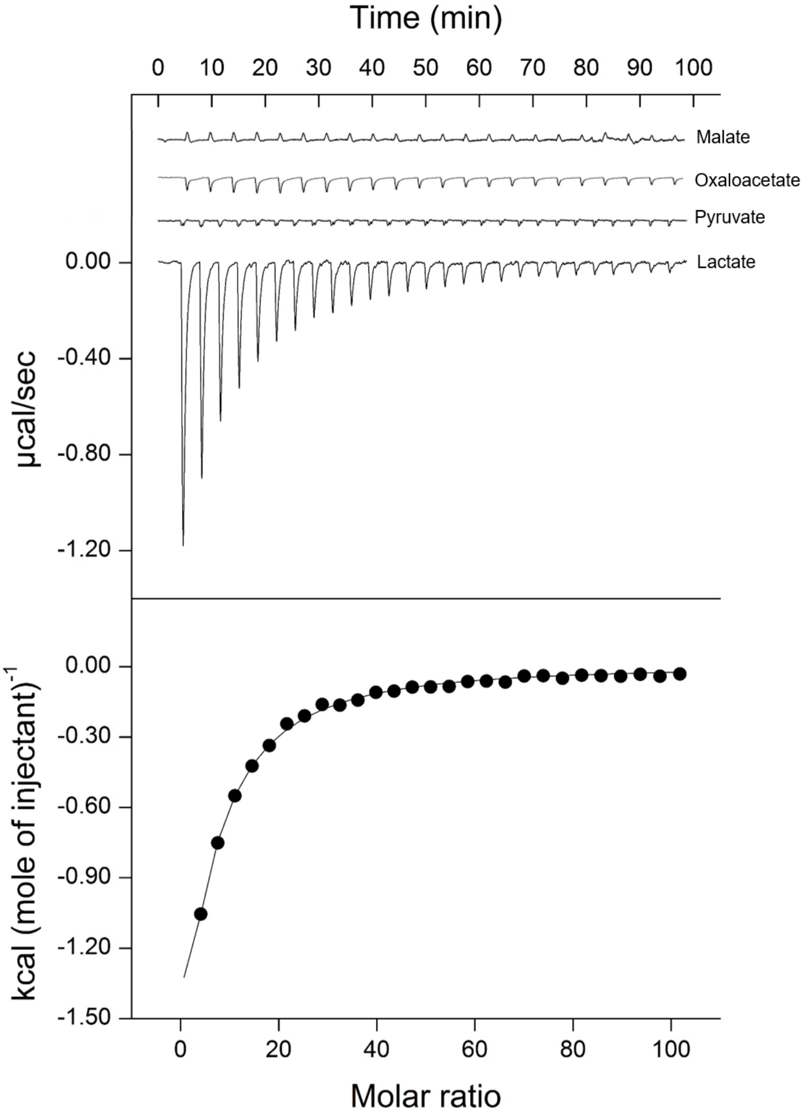

Supplementary Figure 2. ITC titrations of TlpC LBD with malate, oxaloacetate, pyruvate and lactate. Upper panel: raw titration data from the injections of 10 µl of 5 mM ligand solution into a 1.45-ml reaction cell containing 10 µM protein. Lower panel: the integrated and dilution-corrected peak areas of the titration plot for lactate.

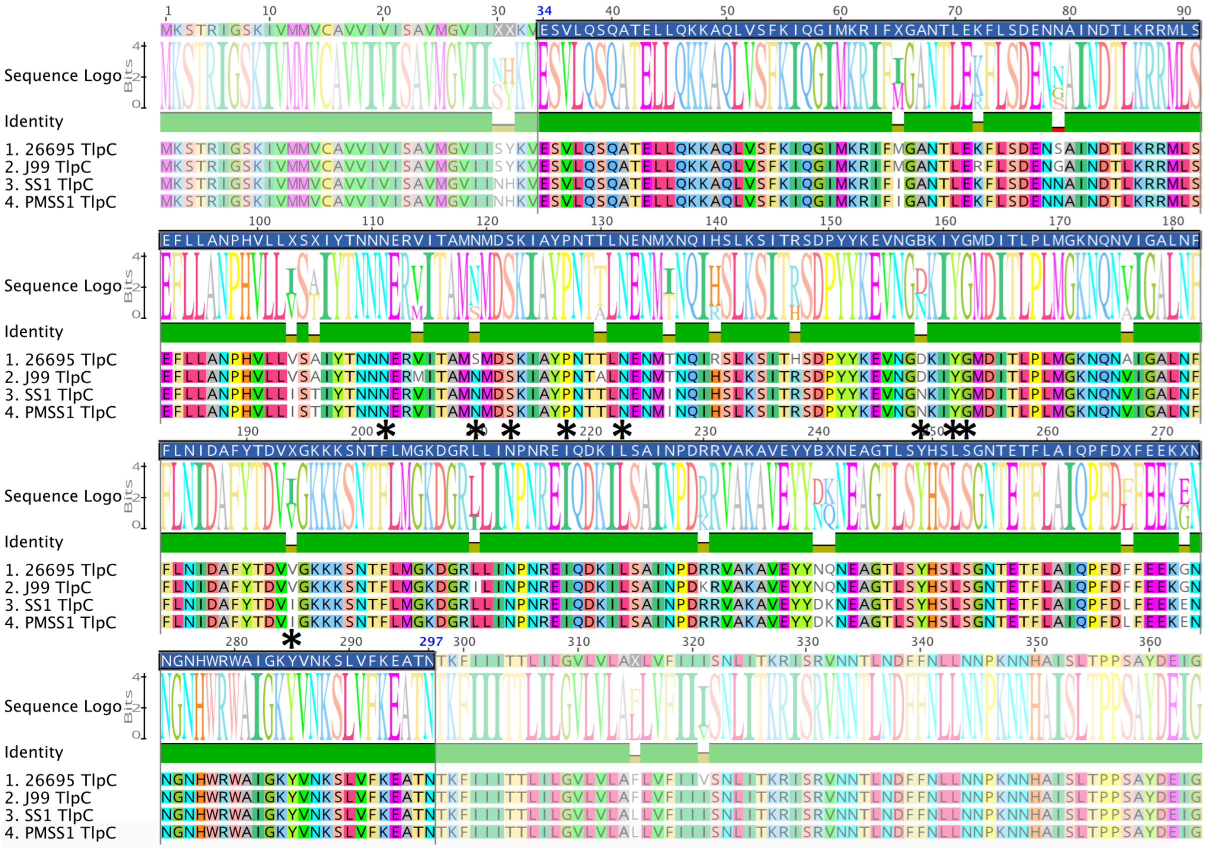

Supplementary Figure 3. Alignment of TlpC protein sequences from *H. pylori* strains 26695, J99, SS1, and PMSS1. Alignments were produced from whole protein sequences using Geneious version 9.1.8 (Biomatters Ltd) with the Geneious alignment function. Highlighted regions show the ligand binding portion of TlpC that was crystallised. Asterisks indicate the residues of the lactate binding pocket, which are all conserved between 26695 and PMSS1. There is one conservative substitution in the J99 TlpC sequence with L210 being changed to I.
