# Supplementary figures and images for: Helicobacter pylori chemoreceptor TlpC mediates chemotaxis to lactate
Source: Sci Rep. 2017 Oct 26;7:14089. doi: 10.1038/s41598-017-14372-2 (PMC5658362; doi:10.1038/s41598-017-14372-2)

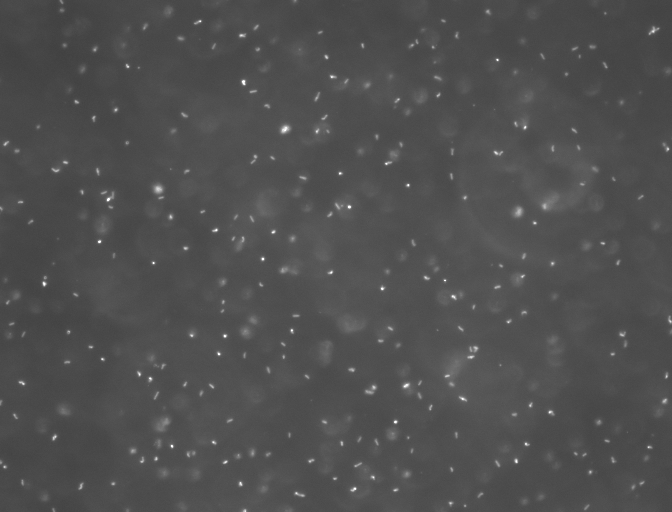

Supplement: Supplementary file 2 — Supplementary video 1 [file 41598_2017_14372_MOESM2_ESM.gif]

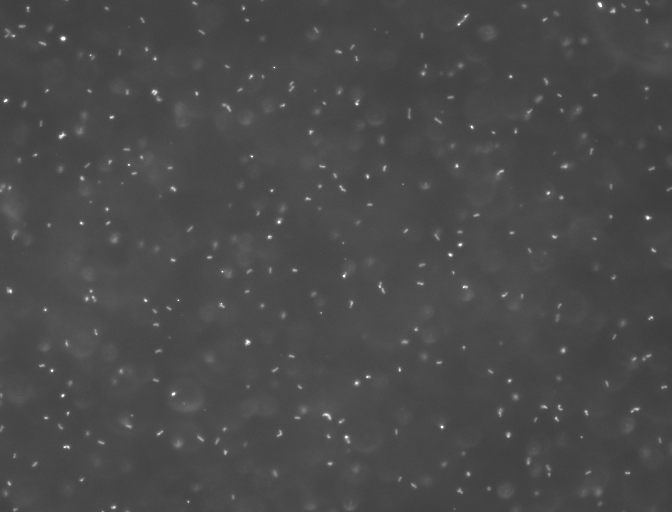

Supplement: Supplementary file 3 — Supplementary video 2 [file 41598_2017_14372_MOESM3_ESM.gif]

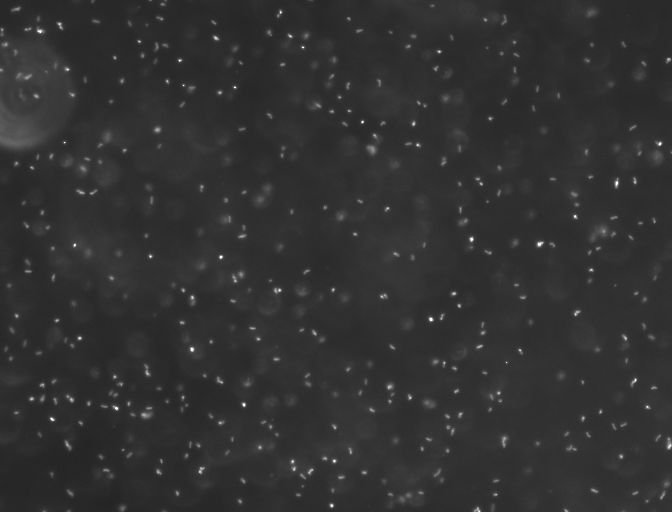

Supplement: Supplementary file 4 — Supplementary video 3 [file 41598_2017_14372_MOESM4_ESM.gif]

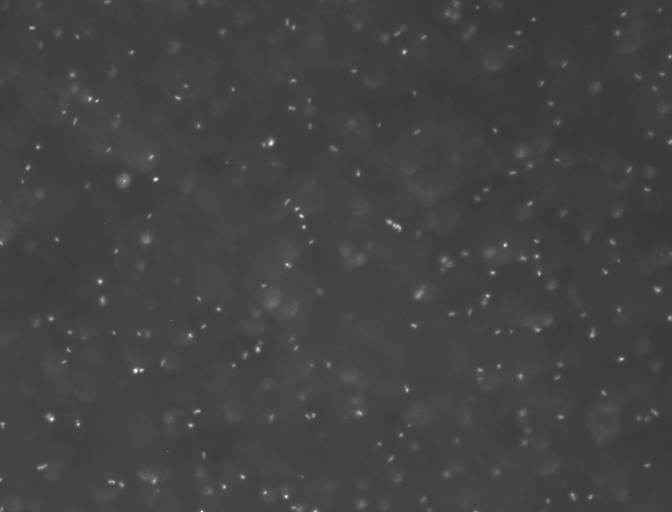

Supplement: Supplementary file 5 — Supplementary Video 4 [file 41598_2017_14372_MOESM5_ESM.gif]

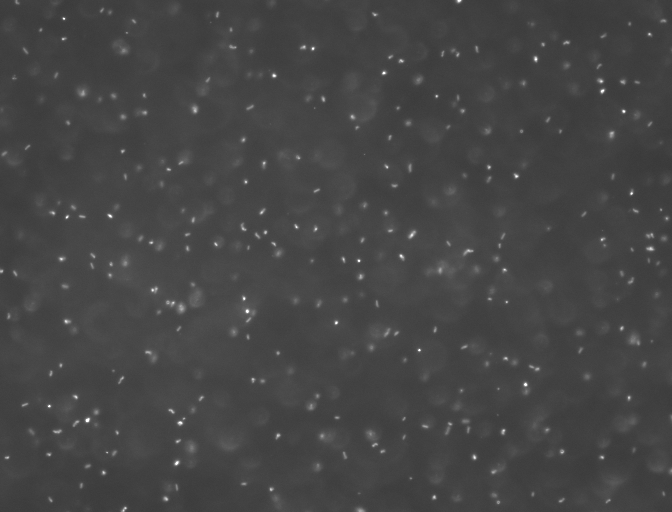

Supplement: Supplementary file 6 — Supplementary video 5 [file 41598_2017_14372_MOESM6_ESM.gif]

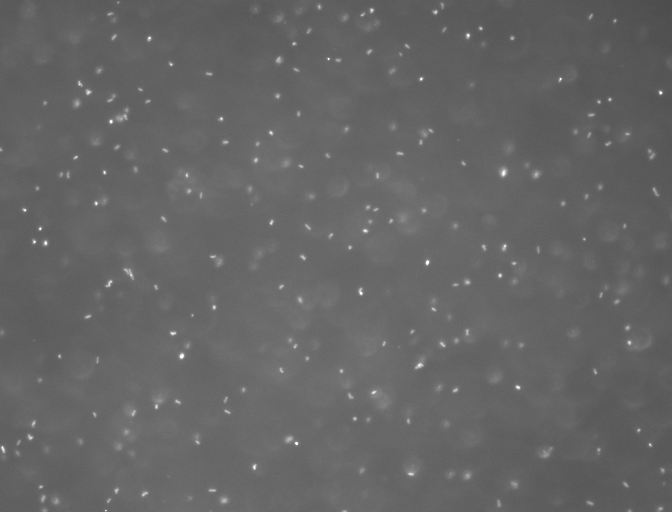

Supplement: Supplementary file 7 — Supplementary video 6 [file 41598_2017_14372_MOESM7_ESM.gif]

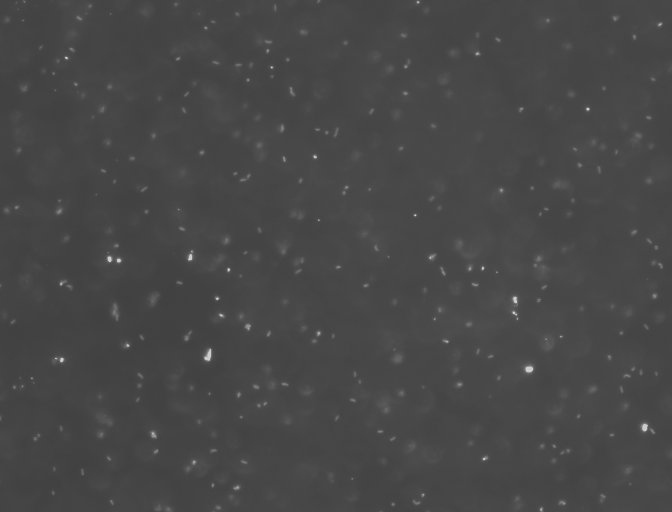

Supplement: Supplementary file 8 — Supplementary video 7 [file 41598_2017_14372_MOESM8_ESM.gif]

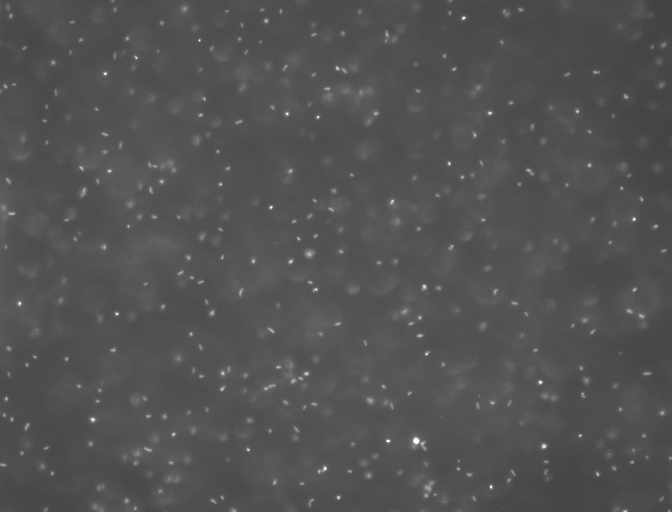

Supplement: Supplementary file 9 — Supplementary video 8 [file 41598_2017_14372_MOESM9_ESM.gif]

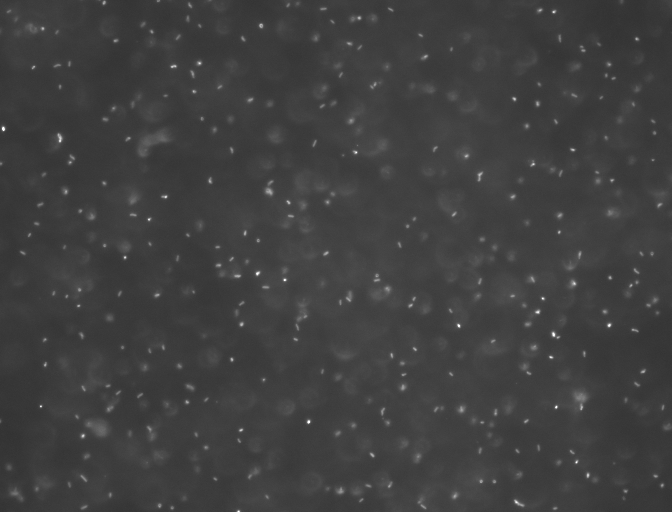

Supplement: Supplementary file 10 — Supplementary video 9 [file 41598_2017_14372_MOESM10_ESM.gif]

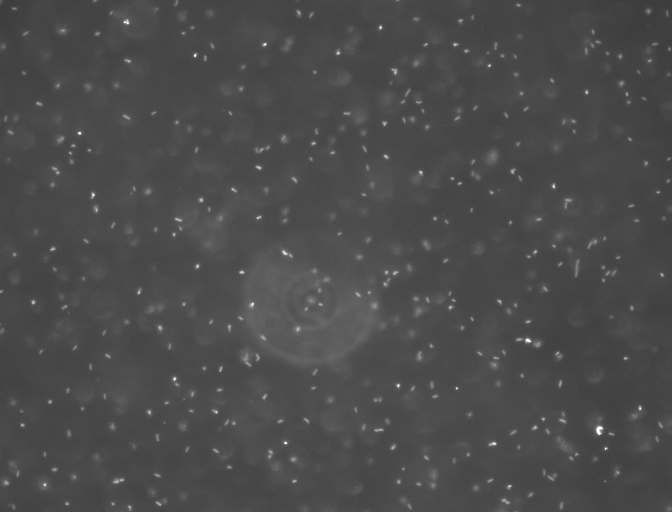

Supplement: Supplementary file 11 — Supplementary video 10 [file 41598_2017_14372_MOESM11_ESM.gif]

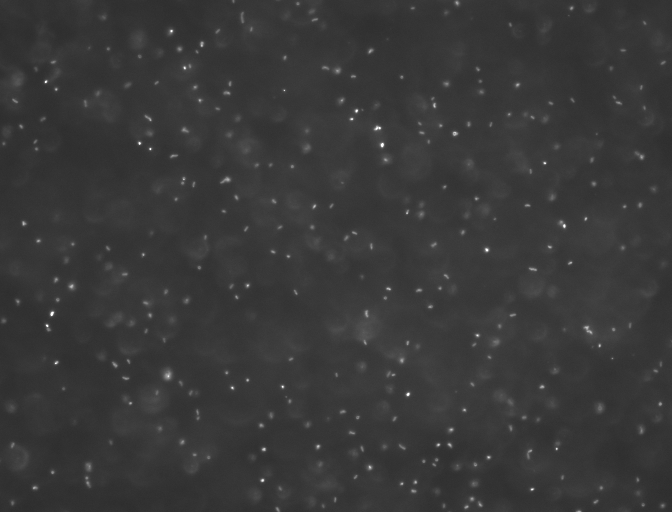

Supplement: Supplementary file 12 — Supplementary video 11 [file 41598_2017_14372_MOESM12_ESM.gif]

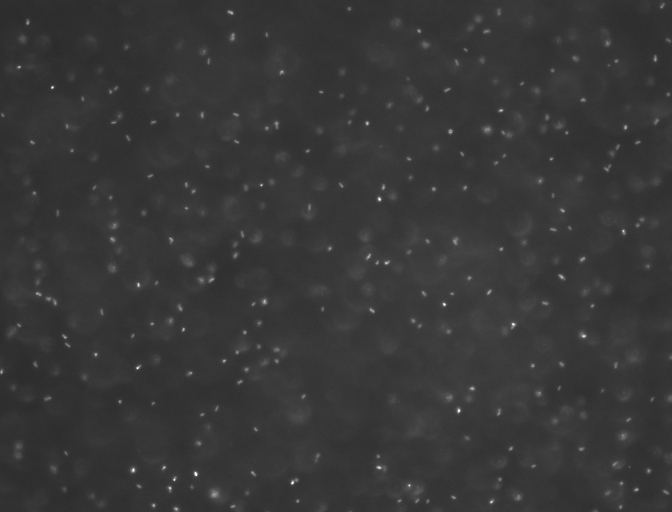

Supplement: Supplementary file 13 — Supplementary video 12 [file 41598_2017_14372_MOESM13_ESM.gif]
